# SARS-CoV-2: An Overview of Virus Genetics, Transmission, and Immunopathogenesis

**DOI:** 10.3390/ijerph18126312

**Published:** 2021-06-10

**Authors:** Mohamed A. Farrag, Haitham M. Amer, Rauf Bhat, Maaweya E. Hamed, Ibrahim M. Aziz, Ayman Mubarak, Turki M Dawoud, Sami G Almalki, Fayez Alghofaili, Ahmad K. Alnemare, Raid Saleem Al-Baradi, Bandar Alosaimi, Wael Alturaiki

**Affiliations:** 1 Department of Botany and Microbiology, College of Science, King Saud University, Riyadh 11451, Saudi Arabia; mfarrag@ksu.edu.sa (M.A.F.); rauf012@yahoo.com (R.B.); mhamed1@ksu.edu.sa (M.E.H.); iaziz@ksu.edu.sa (I.M.A.); aymubarak@ksu.edu.sa (A.M.); tdawoud@ksu.edu.sa (T.M.D.); 2 Department of Virology, Faculty of Veterinary Medicine, Cairo University, Giza 12211, Egypt; hamoamer@cu.edu.eg; 3 Department of Medical Laboratory Sciences, College of Applied Medical Sciences, Majmaah University, Majmaah 11952, Saudi Arabia; Sg.almalki@mu.edu.sa (S.G.A.); f.alghofaily@mu.edu.sa (F.A.); r.albaradie@mu.edu.sa (R.S.A.-B.); 4 Otolaryngology Department, College of Medicine, Majmaah University, Majmaah 11952, Saudi Arabia; a.alnemare@mu.edu.sa; 5 Research Center, King Fahad Medical City, Riyadh 11525, Saudi Arabia; balosaimi@kfmc.med.sa

**Keywords:** coronavirus, COVID-19, cross-species transmission, evolution, immune response, SARS-CoV-2

## Abstract

The human population is currently facing the third and possibly the worst pandemic caused by human coronaviruses (CoVs). The virus was first reported in Wuhan, China, on 31 December 2019 and spread within a short time to almost all countries of the world. Genome analysis of the early virus isolates has revealed high similarity with SARS-CoV and hence the new virus was officially named SARS-CoV-2. Since CoVs have the largest genome among all RNA viruses, they can adapt to many point mutation and recombination events; particularly in the spike gene, which enable these viruses to rapidly change and evolve in nature. CoVs are known to cross the species boundaries by using different cellular receptors. Both animal reservoir and intermediate host for SARS-CoV-2 are still unresolved and necessitate further investigation. In the current review, different aspects of SARS-CoV-2 biology and pathogenicity are discussed, including virus genetics and evolution, spike protein and its role in evolution and adaptation to novel hosts, and virus transmission and persistence in nature. In addition, the immune response developed during SARS-CoV-2 infection is demonstrated with special reference to the interplay between immune cells and their role in disease progression. We believe that the SARS-CoV-2 outbreak will not be the last and spillover of CoVs from bats will continue. Therefore, establishing intervention approaches to reduce the likelihood of future CoVs spillover from natural reservoirs is a priority.

## 1. Introduction

Coronaviruses (CoVs) have long been known to infect human and animals causing a variety of diseases that affect the respiratory airways, intestine, liver, heart, kidneys, and nervous system [1]. Till the beginning of the current millennium, four CoVs (HCoV-229E, HCoV-NL63, HCoV-OC43 and HCoV-HKU1) were described in humans with mild and self-limiting upper respiratory disease. In the last two decades, the world population had witnessed three outbreaks of CoVs with severe respiratory and systemic outcomes, and considerable death rates. 

The first outbreak developed in China in 2002 and spread to several countries worldwide. According to the World Health Organization (WHO), the outbreak that was caused by SARS-CoV-1 affected 8096 cases in 29 countries and costed 774 (9.56%) lives. In 2012, another outbreak caused by MERS-CoV was started in Saudi Arabia, rapidly distributed in the neighboring countries, and further to geographically distant regions [2]. The MERS-CoV outbreak is still ongoing, resulting in a sum of 2519 laboratory-confirmed cases and 866 (34.3%) deaths. 

In December 2019, a third outbreak began in Wuhan, China, and was termed later by WHO as “coronavirus disease 2019” (COVID-19) [3]. During the last few months, the disease spanned the globe and affected more than 141 million with about 3 million (2%) losing their lives [4]. The outbreak was caused by SARS-CoV-2, which is highly antigenically and genetically similar to SARS-CoV-1 [3]. Both SARS-CoV-1 and SARS-CoV-2 share some features; (i) they use the same cellular receptor ACE2, (ii) their internal fusion peptides of the S protein are identical, (iii) they have the same genomic organization with 79.5% sequence similarity. However, a number of dissimilarities were reported, such as the length of the S protein which is longer in SARS-CoV-2, and the presence of additional furin cleavage site in the S protein of SARS-CoV-2. In addition, both viruses show somehow different patterns of clinical presentation, pathogenesis, and epidemiology [5]. SARS-CoV-2 is mainly transmitted among human populations by inhalation of aerosol droplets and touching contaminated surfaces [6]. The disease symptoms are generally diverse; ranging from mild to life-threatening. Although the virus affects all age groups, people with high risk (for example, the elderly, immunosuppressed individuals, and patients with comorbidities) may get more severe disease outcomes and require hospitalization and intensive care facilities [7]. As a consequence, unprecedented measures and restrictions have been adopted to control the spread of the disease and its impact on health and economy, including lock down, partial/complete curfew, and travel restrictions [8].

Currently, several vaccine formats (mRNA, viral vectors, inactivated, and subunit vaccines) have been approved and more than 1.65 billion doses administered worldwide. In the current review, the following topics are covered: (i) SARS-CoV-2 genetics and evolution, (ii) S protein and its role in virus evolution and adaptation through receptor usage, (iii) possible ways of virus transmission, (iv) the immune response and its role in SARS-CoV-2 pathogenicity.

## 2. SARS-CoV-2: Virus Genetics and Evolution

### 2.1. Taxonomy and Genome Organization 

All CoVs are members of the subfamily *Orthocoronavirinae*, family *Coronaviridae*, and order *Nidovirales*. These viruses have the longest genome (26.4–32.0 kb) amongst all recognized RNA viruses [9,10]. The viral RNA genome, which is non-segmented, positive sense, and single stranded, is encapsulated within a helical nucleocapsid and a virus envelope that carries three glycoprotein projections; spike (S), membrane (M), and envelop (E). According to the international committee on taxonomy of viruses (ICTV), four genera have been described within the subfamily *Orthocoronavirinae* including *alphacoronavirus*, *betacoronavirus*, *gamma-coronavirus*, and *deltacoronavirus* [11]. Members of the first two genera infect mammals (including human), while CoVs of the latter two genera mostly infect birds [12]. 

**Figure 1:**
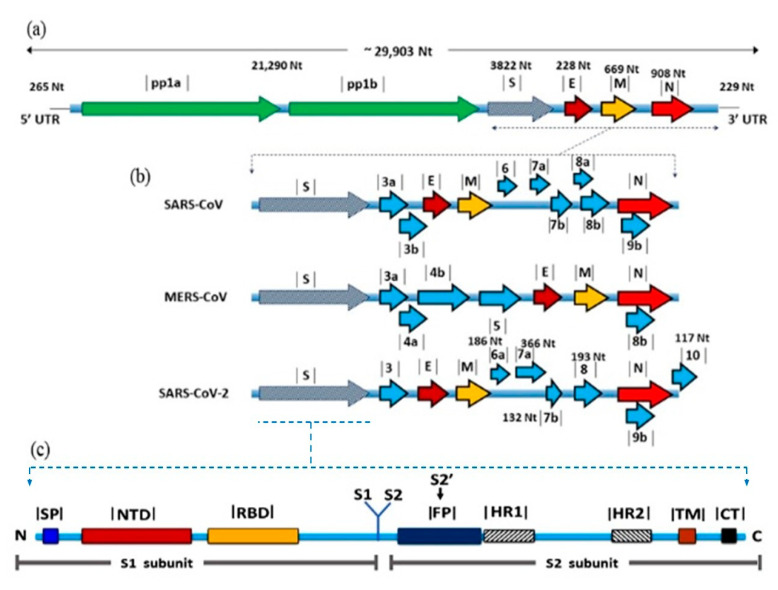
Genomic organization and gene view of three human betacoronaviruses. (a) Coronavirus genome is a positive sense ssRNA, which is flanked by two untranslated regions, 5’ UTR and 3’ UTR. The first two thirds of the genome, ORF1a/b encoding for non-structural proteins required for virus replication. Structural proteins including E, M and N nucleoprotein, and accessory proteins, (**b**) comparison between the three CoVs in terms of the number and positions of accessory proteins, (**c**) detailed structure of the S protein showing the domains involved in receptor binding S1 and membrane fusion S2.

SARS-CoV-2 genome is approximately 29.8 kb long and has a GC content of 38% [13]. The genome is flanked by two untranslated regions (UTRs) and is annotated by 14 open reading frames (ORFs). The first two 5′-ORFs; ORF1ab and ORF1a, extend for about two-thirds of the genome and encodes for 16 non-structural proteins (nsps) that are responsible for virus replication [14]. The last-third ORFs encode for the structural proteins; S, M, E, and nucleocapsid (N), beside eight accessory proteins [15]. The number and location of the accessory proteins are generally variable among CoVs albeit their functions are not fully identified (Figure 1). It is expected that these proteins may play a role in virus replication and pathogenesis [16].

### 2.2. Origin and Evolution

Sequence and phylogenetic analysis of SARS-CoV-2 structural genes have revealed genetic relevance to SARS-CoV-1 (79%) rather than MERS-CoV (50%) [17]. However, several variations enough to clearly discriminate between both SARS-CoVs still exist. For example, ORF8a is absent in SARS-CoV-2 while present in SARS-CoV-1, ORF8b is 38 aa shorter in SARS-CoV-1, and ORF3b is 132 aa shorter in SARS-CoV-2 [18]. SARS-CoV-2 also shares marked sequence homology with bat SARS-Like CoVs from Zhoushan in Eastern China; bat-SL-CoVZC45 (87.99%) and bat-SL-CoVZXC21 (87.23%). Thus, SARS-CoV-2 was allocated in the subgenus Sarbecovirus with the bat coronaviruses BetaCoV/bat/Yunnan/ RaTG13/2013 and SARS-like bat CoVs [14,17]. The level of genetic similarity with RaTG13 is not enough to consider SARS-CoV-2 as a variant; however, bats are thought to be the natural reservoir where SARS-CoV-2 was originated and spread through an intermediate host [19]. 

The intermediate host of SARS-CoV-2 is still an enigma, in contrast to SARS-CoV-1 and MERS-CoV in which their intermediate hosts were resolved as palm civet [20], and dromedary camel [21], respectively. Determining the intermediate host is crucial to stop introduction of viruses to the community by blocking the interspecies transmission. Mass slaughtering of palm civets had a great impact on the termination of SARS-CoV-1 epidemic [20]. 

Early in the pandemic, the virus was thought to spread from a seafood market; however, some patients had no history of visiting these markets [6]. Later on, pangolin was suspected as the intermediate host since highly relevant CoVs were isolated from two dead Malayan pangolins [22]. Complete genome sequences of pangolin CoVs were aligned with different CoVs and confirmed high sequence identity with SARS-CoV-2 (91.02%) and bat RatG13 (90.55%) [23]. The susceptibility of a wide array of laboratory animals, domestic animals, and birds to SARS-CoV-2 was further tested. The virus did not efficiently replicate in birds (chickens and ducks), dogs, and pigs, whereas ferrets and cats were permissive to airborne infections [24]. 

SARS-CoV, as a typical RNA virus, generates quasi species of viruses during replication as a result of the polymerase errors [25]. Consequently, the virus progeny acquires new genetic and antigenic features that improve fitness in the environment and escape from the pre-existing immunity. Several mutations and recombination sites were recognized throughout the genome of SARS-CoV-2. Most importantly, six potential recombination sites identified in S gene were assumed to be responsible for evolutionary survival and adaptation in humans [19]. An 81-nucleotide deletion (27 aa) was defined in ORF7a, which encodes a protein induces apoptosis, and counteracts the action of BST-2 to facilitate virus release during replication [26,27,28]. Similarly, a 382-nucleotide deletion was observed in ORF8 [29]. The impact of this deletion was previously investigated in SARS-CoV-1, and demonstrated reduced virus replication in vitro [30]. In case of SARS-CoV-2, ∆382 nucleotide variants were associated with milder infections [31]. Interestingly, several variants with mutations in the S protein emerged, such as P1, B.1.1.7, B.1.351, B.1.427, and B.1.429. Such variants are associated with different clinical presentation, reinfection rate, and transmissibility [32,33,34]. In addition, some of the current vaccines were found to be less effective against one or more of these variants [35,36].

## 3. S Protein: Role in Virus Evolution and Cross-Species Transmission

CoVs have a great potential to jump between species. Numerous host-switching episodes were reported in the last three decades, including animal-to-human, human-to-animal, and animal-to-animal transmission [20]. Such cross-species transmission had resulted in the development of significant diseases in man and animals [37,38,39,40]. The most evident zoonotic transmission of CoVs involved the emergence of SARS-CoV-1, MERS-CoV, and the pandemic SARS-CoV-2. S protein of CoVs is believed to play a vital role in cross-species transmission [41]. 

S protein is a group I glycoprotein that forms the long projections decorating the surface of CoV particles in a characteristic crown-like shape. It is responsible for initiation of the infection process through binding to specific cell receptors and fusion-mediated entry in host cell [41]. The length of S protein varies among CoVs from 1100 to 1600 amino acids, and the molecular weight is typically around 220 kDa. The primary form of S protein is composed of three defined regions; large ectodomain, transmembrane domain, and cytoplasmic tail (Figure 1b). The ectodomain has two functional domains S1 and S2, which are responsible for receptor binding and membrane fusion, respectively [41,42]. S1 is further subdivided into two subdomains; S1-CTD, which binds to protein receptors [43], and S1-NTD that binds to sugar receptors [40]. After biosynthesis, S proteins are arranged in homotrimers, which exist either in prefusion or post-fusion forms [41]. The prefusion form is metastable and proteolytically cleaved at S1/S2 boundary by various triggers (Table 1) to form the postfusion form [41]. The fact that some host cell proteases are tissue-specific may contribute to CoV tropism to the respiratory tract.

Three main features of S protein contribute to the evolution of CoVs including: (1) the outstanding ability of receptor usage, (2) the high mutation rate, (3) the potential of acquiring new proteolytic cleavage sites. No general receptors were described for CoVs; instead, they use numerous receptors to bind with target cells. Interestingly, CoVs from different genera can use the same receptor and CoVs of the same genus may use different receptors. The high mutation rate of S protein is an expected consequence of its role in cell binding, which makes S protein a major target for immune pressure [41]. A single amino acid change in the receptor-binding domain can lead to host-shifting and development of serious epidemics. For example, two amino acid changes, T31K and E38D, in S1-CTD subdomain of SARS-CoV-1 were predicted to strengthen the binding to human ACE2 receptor. Such changes are potentially the cause of cross-species transmission of SARS-CoV-1 from civets to man [55]. 

Cleavage of S protein and release of the fusion peptide is a crucial step for virus entry. Acquiring new cleavage sites, particularly furin, enhances infectivity and increases the likelihood of systemic disease in many virus infections, such as influenza, Ebola, AIDS, Zika, dengue, and MERS [56,57,58]. The in vitro studies showed that the more furin cleavage sites acquired on S protein of infectious bronchitis (IBV) CoV, the broader host range and the higher ability to infect more cell lines [59]. IBV with new cleavage site on S protein displayed high pathogenicity in chickens [60]. The S protein of SARS-CoV-2 has an additional furin cleavage site that is absent in SARS-CoV-1 [53]. The existence of such new site may enhance virus transmissibility, host range, and the ability to cause systemic infection [61].

S protein of SARS-CoV-2 specifically binds to ACE2 receptors as a prerequisite of cell entry [53]. With the continuous and rapid flow of data, several questions have arisen to help understanding the impact of this process on virus infectivity. For example; (i) what is the degree of sequence similarity between S protein of SARS-CoV-1 and SARS-CoV-2, (ii) do both viruses have the same binding affinity to ACE2. On sequence level, S protein of SARS-CoV-2 shares 98% and 70% sequence identity with its counterparts in RaTG13 and SARS-CoV-1, respectively. Although both SARS-CoVs bind to the same receptor, their S1-CTD subdomains display different antigenic properties. The monoclonal antibodies bind to S1-CTD and the polyclonal antibodies bind to RBD of SARS-CoV-1, failing to recognize their counterparts in SARS-CoV-2 [61]. In contrary, the polyclonal antibodies raised against the complete S protein of SARS-CoV-1 were able to neutralize the infectivity of SARS-CoV-2 [53]. It is worthwhile to mention that S2 domain of both viruses shares high sequence identity (~90%) and many neutralizing epitopes [61]. The binding affinity of S protein of SARS-CoV-2 to ACE2 receptor is generally weaker than the binding of SARS-CoV-1 [3,53,62]. In contrast to SARS-CoV-1 [63], the RBD of SARS-CoV-2 is less accessible due to its laying down dynamic state [53].

## 4. Transmission of SARS-CoV-2

**Figure 2:**
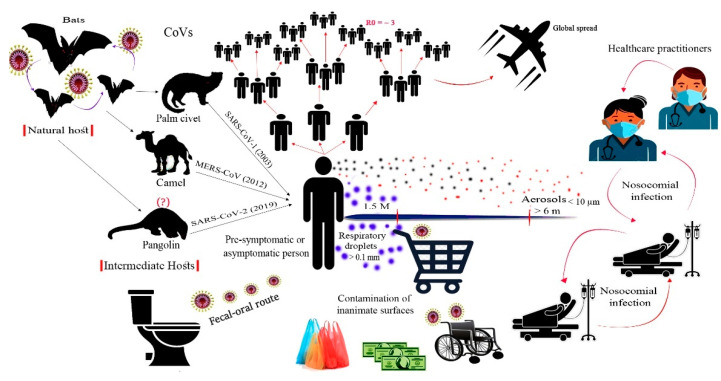
Origin and transmission of SARS-CoV-2. The virus is believed to be originated from bats and spread to humans through an intermediate host. Human-to-human transmission mainly occurred by respiratory aerosols generated when an infected person sneezes or speeches. Respiratory aerosols can travel to long distances, whereas respiratory droplets settle down on nearby surfaces and represent a possible risk of infection. Basic reproduction number (R0) of the virus is estimated as 3.6. The faecal-oral route of transmission was reported due to the ability of the virus to replicate in the gastrointestinal tract. Within hospital settings, nosocomial infection is also known to occur among healthcare practitioners and hospitalized patients.

Similar to other respiratory viruses, SARS-CoV-2 is mainly transmitted via respiratory droplets, aerosols, and close contact with infected persons [5]. The contaminated respiratory droplets are generated during speech and sneezing of infected persons. Large droplets do not stay airborne for a long time and settle down on surfaces, whereas, small droplets and aerosols are carried away and stay airborne for hours [64,65,66]. Aerosols have also been generated in hospitals during bronchoscopy, endotracheal intubation, cardiopulmonary resuscitation, and nebulization (Figure 2) [14,64,65]. The large respiratory droplets represent a primary source of infection either by inhalation prior to settling down or by touching contaminated objects. Airborne transmission is the common route of transmission in enclosed settings with inadequate ventilation (for example, public transportation vehicles, classrooms, offices, restaurants, etc.) [67].

The other transmission routes that should not be neglected involve the fecal-oral and fecal-respiratory routes. Transmission via these routes was previously reported for SARS-CoV-1 and MERS-CoV [68]. SARS-CoV-2 was detected in stool and urine samples of laboratory-confirmed cases, and the virus was isolated from intestinal contents and anal swabs [69]. Interestingly, the virus was detected in these samples after respiratory clearance, which may indicate the prolonged shedding of SARS-CoV-2 from alternative routes, even in patients with negative respiratory samples (Figure 2). In hospital-based patient care settings, high bioaerosol concentrations from flushing fecal wastes have been recorded [70]. These bioaerosols might lead to surface contamination and fecal-respiratory exposures among patients and healthcare workers. 

At the moment, there is no firm evidence that vertical transmission of SARS-CoV-2 occurs; however, the possibility should not be completely omitted. Trials for identification of SARS-CoV-2 RNA on the objects of conception and newborns were all negative [68]. Nonetheless, more recent studies have suggested potential trans-placental transmission of SARS-CoV-2 due to the presence of specific IgG and IgM antibodies [71] and viral RNA in samples collected from newborns at days 2 and 4 post-delivery [72]. 

Pre-symptomatic and asymptomatic infected individuals are important sources of SARS-CoV-2 transmission. Virus transmission from asymptomatic cases was estimated to cause 86.2% of all reported cases worldwide [65]. Globally, it was estimated that around two-thirds of COVID-19 cases exported from China have remained undetected [73]. Therefore, extensive SARS-CoV-2 screening and early detection of asymptomatic cases would impact the transmission efficiency and disease spread in different communities [65].

Persistence of SARS-CoV-2 in the environment; particularly on inanimate objects, has created a state of fear and insecurity in the public [74]. In general, most CoVs can persist in the environment up to one month depending on the surface type. SARS-CoV-2 loses its infectivity after 3 h on printing and tissue papers, 24 h on cardboard, and 2 days on wood and cloth. The virus is more stable on smooth surfaces and can persist up to 4 days on glass and banknote, and 7 days on stainless steel and plastic [64,75]. SARS-CoV-2 is also sensitive to increased temperature and common household disinfectants (for example, hand soap). The virus can withstand temperatures of 4 °C, 22 °C, 37 °C, 56 °C, and 70 °C for 2 weeks, 7 days, 1 day, 10 min, and 5 min, respectively. Interestingly, the virus is stable at a wide range of pH (3–10) [75]. 

Therefore, precautions should be considered by individuals and authorities to reduce virus transmission in the community. Individuals should adopt strict personal hygiene, such as frequent hand washing and mask wearing. Within indoor settings, improved ventilation and avoiding gatherings are necessary. Appropriate biosecurity measures should also be applied in toilets to reduce virus transmission via fecal-oral and fecal-respiratory routes. These measures include closing toilet lids before flushing, keeping toilet fans running, and using the autorun hang washer. 

## 5. Immune Response against SARS-CoV-2

Little knowledge is currently available on the host immune response against SARS-CoV-2 and the immunopathogenesis during COVID-19. Most of the assumptions are based on the available data about SARS-CoV-1 and MERS-CoV [76]. The aggressiveness and disease severity witnessed in SARS-CoV-2 infected individuals, even though in a limited percentage of infected individuals, result from the efficient immune evasion strategies employed by SARS-CoV-2 [77]. It is known that the host antiviral response relies on type I interferon expression of host cells [78]. However, SARS-CoV-1, and most likely SARS-CoV-2, effectively inhibits the interferon response by suppressing activation of the TNF receptor-associated factors (TRAF) 3 and 6, and the transcription factors NFκB, IRF3, IRF7, and reducing secretion of the pro-inflammatory effector cytokines IL-1, IL-6 and TNF-α [79]. Inhibiting type 1 IFN signaling suppresses anti-viral programs, while increasing IL-1, IL-6, and TNF-α expression amplifies hyperinflammation and cytokine storm through positive feedback [77,79]. Altogether, suppression of innate immune mechanisms in infected cells allows SARS-CoV-2 to proliferate without triggering the innate antiviral response machinery.

**Figure 3:**
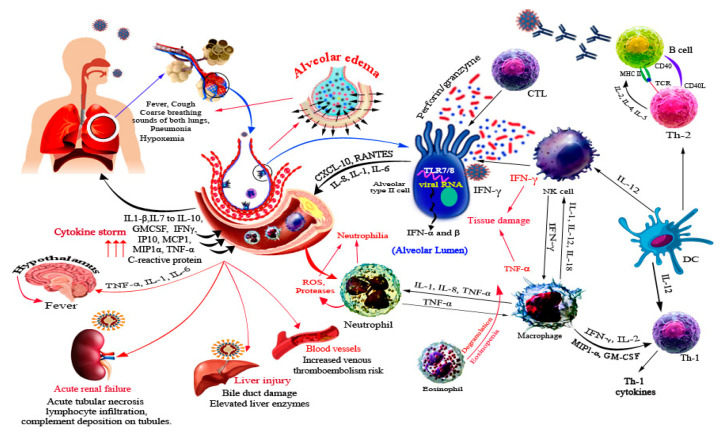
Immune response and immunopathogenesis associated with SARS-CoV-2. SARS-CoV-2 infects mainly type II alveolar cells and upon infection a wide array of cytokines and chemokines is secreted, which recruits several immune innate cells. Recruitment of cells further potentiates the immune response and usually results in tissue damage due to high concentration of IFN-γ and TNF-α. In addition, degranulation of eosinophils, proteases and ROS of neutrophils cause tissue damage. The participation of immune response in disease progression is illustrated by red arrows. SARS-CoV-2 is reported to cause systemic infections of liver, kidneys, and blood vessels.

The host immune response to SARS-CoV-2 is not only directed against the virus but also involves immune dysregulation and hyperactivity [80]. SARS-CoV-2 infection initiates a systemic immune response recruiting inflammatory macrophages and monocytes, cytokine production, and priming adaptive T and B cell immune responses (Figure 3). In the majority of cases, the infection resolves on its own, whereas a dysregulated and excessive immune response occurs in some cases, causing severe lung disease and systemic pathology (Figure 3) [6]. 

The cascade of host response against SARS-CoV-2 is initiated by tissue resident alveolar epithelial cells and macrophages by activation of host pattern-recognition receptors (PRRs) that detect the released virus-associated molecular patterns (PAMPs). This leads to a burst of local inflammation releasing pro-inflammatory cytokines and chemokines such as IL-6, IFN-γ, monocyte chemoattractant protein-1 (MCP-1), IL-2, IL-7, IL-10, granulocyte colony-stimulating factor (GM-CSF), IP-10, macrophage inflammatory protein 1α (MIP1-α), and tumor necrosis factor (TNF) [6,81]. These cytokines cause a T helper 1 (TH1) cell-polarized response by attracting immune cells, notably CD14+CD16+ inflammatory monocytes and T lymphocytes, from blood towards the infection site [6,82]. Uncontrollable inflammatory cell infiltration may lead to excessive lung damage through disproportionate release of proteases, reactive oxygen species, and TNF-α causing septic shock and multi-organ failure (Figure 3) [82]. 

Clinical data of 128 cases in Iran has shown an enhanced number and activity of CD8+ T cells rather than CD4+ T cell responses [83]. The detected virus-specific T cells were mostly central memory phenotype with high frequency of polyfunctional CD4+ and CD8+ T cells secreting IL-2, IFN-γ, and TNF-α. As a result of pro-inflammatory cytokine expression and the presence of nuclear antigens, T cells may trigger a second burst of inflammation at late disease stages [83,84]. 

Recent data from China demonstrated rapid reduction of T lymphocytes (both CD4+ and CD8+), HLA-DR, and CD38 double-positive fractions in the peripheral blood of SARS-CoV-2 infected patients. Moreover, there was an increased percentage of proinflammatory CCR6+ Th17 cells and an increased concentration of cytotoxic granules in CD8+ T cells [84]. Another group has reported that CD4+ T cells produced less IFN-γ in severe cases with no change in CD8+ T and NK cells [85]. C45RA+ naïve Tregs and memory CD45RO+ memory Tregs were found in low and high proportions, respectively. On recovery, there was a rapid restoration of CD3+, CD4+, and CD8+ T cells along with B and NK cell counts within 2–3 months [85]. Taken together, these changes in lymphocyte populations suggest dramatic dysregulation, evidence of T cell “exhaustion”, and shifts in the adaptive immune response to SARS-CoVs [84]. SARS-CoV-2 infected patients have also shown high levels of IL-1 beta, IFN-γ, IP-10, and MCP-1, which probably leads to activated T-helper-1 cell response and cytokine storm [85]. However, SARS-CoV-1 has been shown to induce pro-inflammatory and NK cells more significantly than SARS-CoV-2, which may explain the higher severity and mortality of the former [86].

Humoral immune response is crucial for virus neutralization and for providing a source of convalescent plasma to treat seriously-ill patients [86]. SARS-CoV-2 infection induces production of IgG against N protein starting from day 4, and IgM on the 9th day of disease onset. The class switching from IgM to IgG was detected in the second week [84]. Early production of neutralizing antibodies may be another factor contributing to organ damage and poor disease outcome. ADE through binding to Fcγ receptors may also contribute to persistent viral replication in immune cells and inflammatory responses causing tissue and organ damage; including acute respiratory distress syndrome (ARDS) [77]. Positive SARS-CoV-2 cases mostly mount an robust IgG antibody response in three or more weeks, which may protect against re-infection [87].

## 6. SARS-CoV-2 Related Organ Pathogenesis

SARS-CoV-2 virus predominantly infects lung epithelial alveolar cells causing severe pneumonia and occasionally leads to ARDS. Lower respiratory tract symptoms also include fever, dry cough, headache, dizziness, general weakness, vomiting, and diarrhea [88]. Autopsy reports of COVID-19 patients have shown diffuse alveolar impairment with hyaline membrane formation and immune cell infiltration in the alveoli [89]. Other organ systems such as kidneys, liver, intestine, heart, spleen, and brain are also affected in the course of SARS-CoV-2 infection and may induce more severe complications.

Acute kidney injury is a life-threating manifestation that may develop in seriously ill patients and often causes death. The potential mechanisms for such injury involve sepsis-causing cytokine storm and direct viral-mediated cellular injury. This may lead to impairment of gaseous exchange and development of secondary infections [89]. The mechanism by which SARS-CoV-2 induces liver injury remains unclear. However, it is predicted that the viral cytopathic effect and/or the immune-pathology induced by dysregulated inflammatory responses may be responsible for hepatomegaly, degeneration of hepatocytes, and inflammatory cell infiltration in liver [90]. 

In many recorded cases, gastrointestinal symptoms including nausea, vomiting, and diarrhea may develop. Some patients also experience altered mental status, reduced urine output, cold extremities, low blood pressure, and blotchy skin [91]. Spleen atrophy is often accompanied by necrosis, macrophage hyperplasia, and significant reduction in lymphocytes. Cardiac infection may trigger degeneration and necrosis of cardiomyocytes, and infiltration of monocytes, lymphocytes, and neutrophils in the myocardial interstitium. Sometimes, the brain demonstrates congestion, edema, and scattered neural-degeneration [88,89,91]. Individual with comorbidities such as kidney dysfunctions, hypertension, cancers, and diabetes mellitus are more prone to severe SARS-CoV-2 infections. Hypertension is the most common underlying disease in SARS-CoV-2 patients, followed by obesity and diabetes. However, chronic kidney disorders are usually associated with death in COVID-19 infected individuals [92]. The increased risk of COVID-19 severity in hypertension and diabetic individuals may be attributed to thrombotic complications [93]. In addition, thiazolidinedione, which is usually prescribed for diabetic patients, increases the expression of ACE2 receptor and thus promotes SARS-CoV-2 replication [92,94], similarly for cancer patients receiving immunosuppressive drugs due to enhanced SARS-CoV-2 replication [92]. 

## 7. Conclusions

SARS-CoV-2 is a newly emerged virus that has caused a major global pandemic in the 21st century with more than 40 million cases and 1 million deaths. The control measures and restrictions adopted have greatly impacted the economies of all countries worldwide. The virus is believed to have originated from bats and spread to humans via an ill-defined intermediate host. Human-to-human transmission principally occurs through direct inhalation of respiratory droplets and by touching contaminated surfaces. Although SARS-CoV-2 mainly affects the lungs, lesions may also develop in kidneys, liver, intestine, spleen, and brain. In many cases, a cytokine storm, which represents an exaggerated immune response against SAES-CoV-2 infection, exacerbates the disease consequence. Available knowledge regarding virus transmission, evolution, and immunopathogenesis is still lacking to provide a concrete basis for establishing proper and effective control strategies. At present, the scientific community is rushing to collect information on the virus and the disease, besides developing potent drugs, vaccines, and methodologies to reduce the extent of the pandemic on human health and economy. 

## Figures and Tables

**Table 1:** Receptor usage and fusion triggers of most important CoVs species.

Genus	Species	Receptor	Fusion Triggers	Reference
Alphacoronavirus	HCoV-NL63	ACE2		[44]
	HCoV-229E	Aminopeptidase N (APN)		[45]
	TGEV			[46]
	PEDV			[47]
	PRCV			[48] [48] [48]
	Feline-CoV			
	Canine-CoV			
Betacoronavirus	MERS-CoV	Dipeptidyl peptidase 4 (DPP4)	Cleaved by proprotein convertases during virus packaging. Cleaved by lysosomal proteases (e.g., cathepsin L and cathepsin B). pH has no direct triggering effect. Extracellular proteases (e.g., elastases) Cell surface proteases (e.g., TMPRSS2)	[49]
	MHV	Carcinoembryonic antigen-related cell adhesion molecule 1	Cleaved by proprotein convertases during virus packaging. Receptor binding.	[50]
	Bovine-CoV	5-N-acetyl-9-O-acetyl neuraminic acid, Neu 5,9 Ac2		[51]
	HCoV-OC43			
	HKU4	Dipeptidyl peptidase 4	Cleaved only by bat lysosomal proteases.	
	SARS-CoV-1	ACE2	Remains intact in the mature virions (i.e., not cleaved during virus packaging) Cleaved by lysosomal proteases (e.g., cathepsin L and cathepsin B). pH has no direct triggering effect. Extracellular proteases (e.g., elastases) Cell surface proteases (e.g., TMPRSS2)	[52]
	SARS-CoV-2			[53]
Gammacoronavirs	IBV	Sugar		[54]

HCoV: Human coronavirus; TGEV: Transmissible gastroenteritis virus; PEDV: Porcine Epidemic Diarrhea Coronavirus; PRCV: Porcine respiratory coronavirus; CoV: Coronavirus; MHV: Murine hepatitis virus, HKU4: Tylonycteris bat coronavirus (Bat-CoV), IBV: Infectious bronchitis virus; ACE2: *Angiotensin-converting enzyme*.

## Data Availability

Data is contained within the article.
